# Comparison of Gut Bacterial Communities of *Grapholita molesta* (Lepidoptera: Tortricidae) Reared on Different Host Plants

**DOI:** 10.3390/ijms22136843

**Published:** 2021-06-25

**Authors:** Xiangqun Yuan, Xuan Zhang, Xueying Liu, Yanlu Dong, Zizheng Yan, Dongbiao Lv, Ping Wang, Yiping Li

**Affiliations:** 1 Key Laboratory of Integrated Pest Management on Crops in Northwestern Loess Plateau, Ministry of Agriculture, Northwest A&F University, Yangling 712100, China; yuanxq@nwsuaf.edu.cn (X.Y.); zhangxuan@nwsuaf.edu.cn (X.Z.); xueyingliu@nwsuaf.edu.cn (X.L.); yanludong@nwsuaf.edu.cn (Y.D.); 2713649881@nwsuaf.edu.cn (Z.Y.); dongbiao_0613@163.com (D.L.); 2 Key Laboratory of Plant Protection Resources and Pest Management, Ministry of Education, Northwest A&F University, Yangling 712100, China; 3 Department of Entomology, Cornell University, Ithaca, NY 14850, USA; pingwang@cornell.edu

**Keywords:** *Grapholita molesta*, gut microbiota, host diet, host adaptation, 16S rDNA

## Abstract

Intestinal symbiotic bacteria have played an important role in the digestion, immunity detoxification, mating, and reproduction of insects during long-term coevolution. The oriental fruit moth, *Grapholita molesta*, is an important fruit tree pest worldwide. However, the composition of the *G. molesta* microbial community, especially of the gut microbiome, remains unclear. To explore the differences of gut microbiota of *G. molesta* when reared on different host plants, we determined the gut bacterial structure when *G. molesta* was transferred from an artificial diet to different host plants (apples, peaches, nectarines, crisp pears, plums, peach shoots) by amplicon sequencing technology. The results showed that Proteobacteria and Firmicutes are dominant in the gut microbiota of *G. molesta*. Plum-feeding *G. molesta* had the highest richness and diversity of gut microbiota, while apple-feeding *G. molesta* had the lowest. PCoA and PERMANOVA analysis revealed that there were significant differences in the gut microbiota structure of *G. molesta* on different diets. PICRUSt2 analysis indicated that most of the functional prediction pathways were concentrated in metabolic and cellular processes. Our results confirmed that gut bacterial communities of *G. molesta* can be influenced by host diets and may play an important role in host adaptation.

## 1. Introduction

All metazoans harbor substantial numbers of commensal microorganisms in the gut [1]. In the process of long-term coevolution, insects and gut microorganisms have formed an interdependent symbiotic relationship. Insects provide a stable living environment and essential nutrients for intestinal microorganisms, which are also involved in a variety of insect metabolic processes, providing some nutrients for insects and digesting complex carbohydrates [2]. It has been widely confirmed that symbiotic microorganisms participate in the metabolism of the host [3,4], degrade exogenous biological toxins [5,6,7], help the host absorb nutrients [8], regulate the host’s adaptability [4,9], protect the host from pathogens [10,11], regulate mating and reproduction [12,13], promote growth and development [14], and affect the transmission efficiency of vector insects [15,16].

*Grapholita molesta*, known as the oriental fruit moth, is an important fruit tree pest widely distributed in the fruit-growing areas of Asia, Europe, America, Australia, and Africa [17,18,19,20]. It has the characteristic of host shifting. Larvae usually harm the tender shoots of peach trees first, which causes the shoots’ dieback. It will then drill into and eat the fruits of peaches, pears, and apples, resulting in fruit shedding, which seriously affects the product and quality of fruits, causing a great loss of economy [19,21,22,23]. The fruit- and shoot-boring habit makes conventional insecticides poorly effective [22]. In addition, the widespread use of conventional insecticides could lead to many problems, for example, resistance to conventional insecticides, environmental pollution, human health impacts, and injury to beneficial insects. *Bacillus thuringiensis*, as a biological agent with insecticidal activity, has been widely used in the pest control of agriculture and forestry. However, the sensitivity of larvae to *B. thuringiensis* varies due to the specificity of insect hosts and the diversity of gut microbiota. Indigenous gut bacteria increased the susceptibility of larvae to *B. thuringiensis* and contributed to *B. thuringiensis* insecticidal activity in *Lymantria dispar*, *Vanessa cardui*, *Manduca sexta*, *Pieris rapae*, and *Spodoptera littoralis* [24,25,26], while in *Galleria mellonella* and *Homona magnanima*, indigenous gut bacteria increased the tolerance of larvae to *B. thuringiensis* toxin [27,28]. Moreover, the resistance of larvae to entomopathogenic bacterium *B. thuringiensis* is also affected by the maturity of the host plant. Research by Martemyanov showed that when larvae feed on mature leaves, the negative effects of *B. thuringiensis* were eliminated [29]. Therefore, intestinal microorganism is an important entry point to reveal the adaptation mechanism between different hosts of *G. molesta*. 

The composition of the gut microbiota of insects can be affected by many factors, such as host species, genotype, diet, and the host living environment. Inhibition of *Caudal* gene expression of *Drosophila* by RNA interference led to overexpression of antimicrobial peptides, which in turn changed the gut bacterial community [30]. The structures of gut microbiota of *G. molesta* and *Carposina sasakii* were significantly different, even when fed with the same Fuji apples and under the same conditions [31]. The 16S rDNA amplicon sequencing of spider mite populations from five different regions in China found that *Flavobacterium*, *Glutamicibacter*, *Bosea, Xanthobacter*, *Acinetobacter*, *Stenotrophomonas*, and *Caulobacter* showed host-species specificity [32]. Yang et al. revealed that the diversity of gut microbes in *Plutella xylostella* was significantly decreased after the host shifted from radishes to peas and was raised for 17 generations [7]. There have been some studies on the gut microbiota of *G. molesta* [20,31,33], but as far as we know, there are few reports about the structure of the gut bacterial communities when *G. molesta* feed on the fruits or young shoots of Rosaceae hosts.

To explore the impact of host plants on the gut communities of *G. molesta*, we used 16S rDNA sequencing to investigate the structure of the gut communities of *G. molesta* fed on artificial diets, apples, pears, nectarines, peaches, plums, and peach shoots. We found that the structure of the gut communities of *G. molesta* changes significantly after transfer from an artificial diet to the four host fruits of apples, pears, nectarines, and peaches and peach shoots, and the abundance of Proteobacteria increases significantly, especially when feeding on apples and peaches. After transfer from an artificial diet to plums, the diversity of the gut communities of *G. molesta* increased. The relative abundance of Firmicutes and Bacteroidota significantly increased, and that of Proteobacteria was decreased. Our results suggest that both the diversity and relative abundance of *G. molesta* gut bacterial communities are influenced by the host plant.

## 2. Results

### 2.1. Analysis of 16S rDNA Sequencing Results 

**Figure 1:**
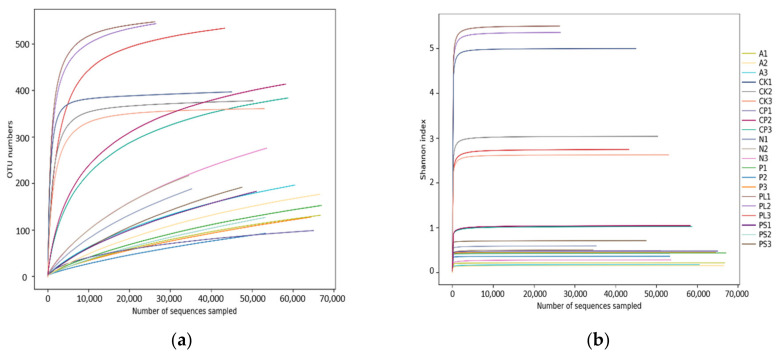
(**a**) Sample rarefaction curves and (**b**) the Shannon index rarefaction curves.

Seven groups of 21 samples were sequenced by Illumina HiSeq 2500 to obtain 1,531,209 pairs of reads. A total of 1,285,740 clean tags were generated after double-ended read stitching and filtering. Each sample generated at least 36,618 clean tags and an average of 61,226 clean tags (Table 1). Cluster analysis (based on 97% sequence similarity) obtained a total of 645 OTUs, including 18 phyla, 30 classes, 80 orders, 127 families, 224 genera, and 292 species. The sample rarefaction curve (Figure 1a) and the Shannon index rarefaction curve (Figure 1b) indicate that the sequencing volume is sufficient and the sequencing depth is saturated and that increasing the sample volume will not produce more OTUs. Meanwhile, we used Good’s coverage to check the completeness of sequencing. The results showed that the coverage of each sample was above 99%, indicating that most species in the sample were identified.

### 2.2. Comparison of the Gut Microbiota between Different Diets

**Figure 2:**
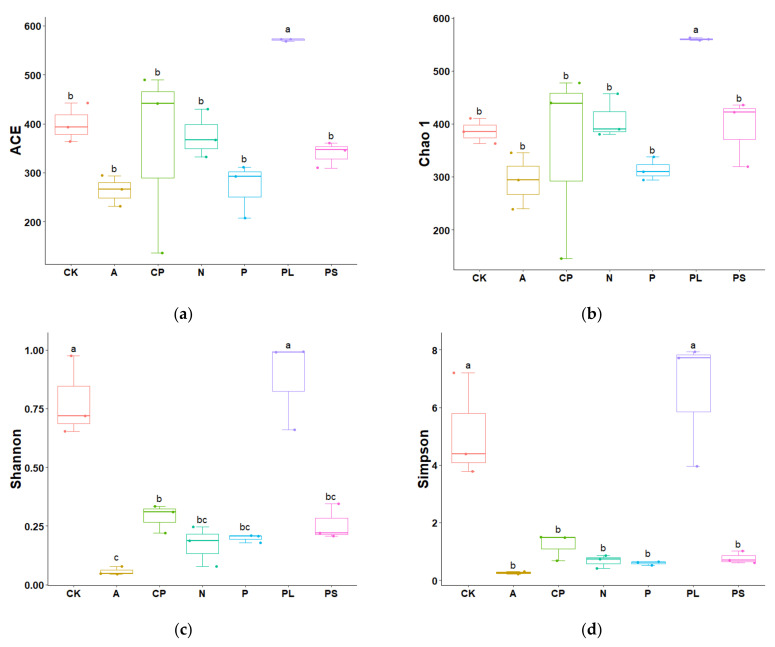
Box plots of (**a**) ACE, (**b**) Chao 1, (**c**) Shannon, and (**d**) Simpson’s values of gut microbiota of *Grapholita molesta* feeding on different host plants. Different capital letters on the abscissa represent different diets: CK, artificial diet; A, apple-feeding; CP, crisp pear-feeding; N, nectarine-feeding; P, peach-feeding; PL, plum-feeding; PS, peach shoot-feeding. Different lowercase letters on boxes indicate significant differences (one-way ANOVA, Tukey post-hoc test, *p* < 0.05) in the mean values.

The alpha diversity index shows that there are differences in the gut microbiota of *G. molesta* feeding on different host plants. The ACE and Chao 1 indices showed that the richness of gut microbiota of the *G. molesta* feeding on plums (PL) was significantly higher than that of other groups. The apple-feeding group (A) had the lowest values in both ACE and Chao 1 (Figure 2a,b). There was no significant difference in Shannon and Simpson’s indices between the *G. molesta* fed on plums (PL) and an artificial diet (CK), but these two groups were significantly higher than that of the other five treatments (*p* < 0.05). Similarly, the apple-feeding group (A) had the lowest values in both Shannon and Simpson’s (Figure 2c,d). Thus, the richness of gut microbiota of *G. molesta* feeding on plums (PL) increased after *G. molesta* was transferred from an artificial diet to plums. Additional the diversity decreased in different degrees after *G. molesta* was transferred from an artificial diet to the other five plants, except plums, especially to apples in Rosaceae.

**Figure 3:**
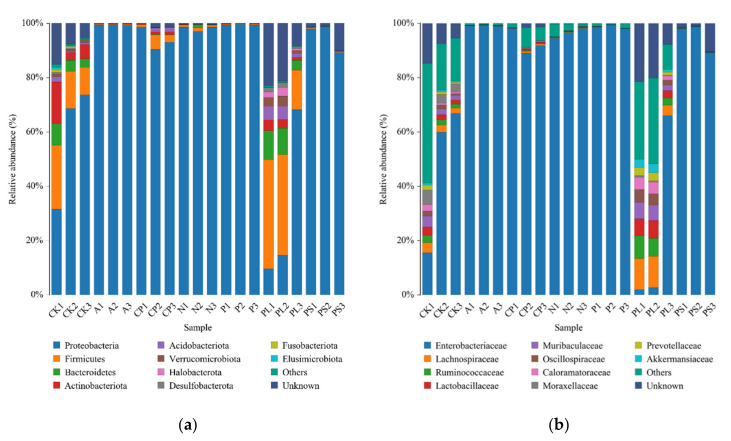
Bacterial composition of the top 10 relative abundances at (**a**) the phylum level and (**b**) the family level. Each color represents a species, and the height of the color block indicates the proportion of the species in relative abundance. Other species are incorporated as “Others” shown in the diagram. “Unknown” represents species that have not received a taxonomic annotation.

We removed the extremely low abundance OTUs with species abundance less than 0.005%, compared the representative sequence of OTUs with the microbial reference database to obtain the species classification information corresponding to each OTU, and then at each level (phylum, class, order, family, genus, species), we counted the composition of each sample community. At the phylum level (Figure 3a), Proteobacteria, Firmicutes, Bacteroidota, Actinobacteriota, Acidobacteriota, Verrucomicrobiota, Halobacterota, Desulfobacterota, Patescibacteria, and Fusobacteriota were the top 10 phyla in relative abundance. Among them, Proteobacteria was the absolute dominant phylum, having the highest relative abundance in the peach-feeding group (P), 99.52 ± 0.12%. The relative abundances in other groups were as follows: 59.18 ± 13.26% in artificial diet-feeding (CK), 99.43 ± 0.07% in apple-feeding (A), 94.34 ± 0.32% in crisp pear-feeding (CP), 98.13 ± 0.60% in nectarine-feeding (N), 32.75 ± 18.59% in plum-feeding (PL), and 95.63 ± 2.93% in peach shoot-feeding (PS). Firmicutes, Bacteroidota, and Actinobacteriota were also dominant in artificial diet-feeding (CK) and plum-feeding groups (PL), and the relative abundances were 17.74 ± 4.85% and 32.92 ± 8.90%, 8.32 ± 2.56% and 15.06 ± 4.06%, and 7.85 ± 3.79% and 2.80 ± 0.86%, respectively (Table 2). At the family level (Figure 3b), Enterobacteriaceae was ubiquitous in most samples. The plum-feeding group (PL) had the lowest relative abundance of Enterobacteriaceae. However, the relative abundances of Lachnospiraceae (8.97 ± 2.53%), Ruminococcaceae (5.86 ± 1.71%), Lactobacillaceae (5.21 ± 1.21%), Muribaculaceae (4.59 ± 1.30%), Oscillospiraceae (3.53 ± 0.87%), Caloramatoraceae (3.50 ± 0.94%), Prevotellaceae (2.39 ± 0.62%), and Akkermansiaceae (2.41 ± 0.69%) in the plum-feeding group (PL) were significantly higher than that in the other six groups (Table 3).

**Figure 4:**
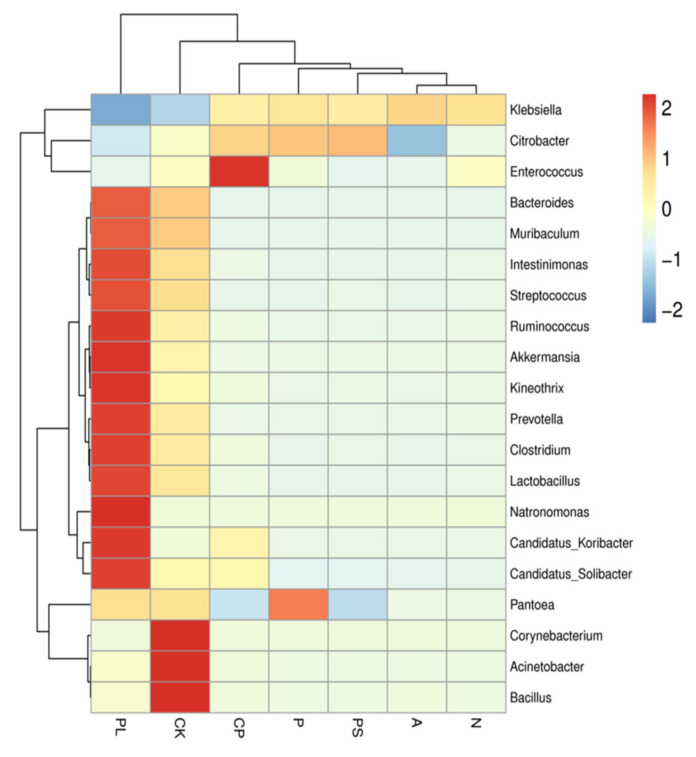
Cluster heat map of the 20 most abundant genera in the bacterial community. The columns represent the samples and the rows represent the bacterial OTUs assigned to the genus level. Dendrograms of hierarchical cluster analysis grouping genera and samples are shown on the left and at the top, respectively.

In order to reveal the dynamic changes in the gut microbiota of *G. molesta* feeding on different host plants, we selected the top 20 genera with relative abundances to draw a relative abundance cluster heat map. Clustering is based on the similarity of species abundance, horizontal clustering is sample information, and vertical clustering is species information. The gut microbiota of *G. molesta* feeding on plums (PL) and an artificial feed (CK) were similar in composition at the genus level; those feeding on crisp pears (CP), peaches (P), peach shoots (PS), apples (A), and nectarines (N) were in different branches. Enterococcus was the main component of the gut microbiota of *G. molesta* feeding on crisp pears, and the relative abundance of *Enterococcus* in the gut bacterial community of *G. molesta* feeding on crisp pears was higher than that for the other groups and the same for *Pantoea* in the peach-feeding group and *Acinetobacter*, *Bacillus*, and *Corynebacterium* in the artificial diet-feeding group (Figure 4).

**Figure 5:**
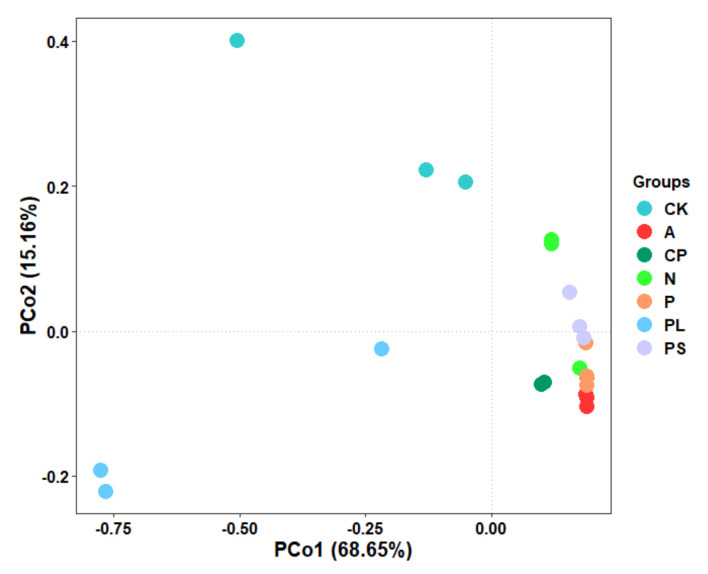
Principal coordinate analysis (PCoA) based on the Bray−Curtis distance between different host diets. CK, artificial diet; A, apple-feeding; CP, crisp pear-feeding; N, nectarine-feeding; P, peach-feeding; PL, plum-feeding; PS, peach shoot-feeding.

The PCoA analysis based on the Bray−Curtis distance was used to compare the community similarities between samples. The PCoA scatter plot showed that the abscissa and ordinate represent the two characteristic values that contribute to the largest differences between the samples, and their influence degrees were 68.65% and 15.16%, respectively (Figure 5). PERMANOVA analyses show that there are significant differences between the different groups (PERMANOVA: R^2^ = 0.787, *p* = 0.001). The structure of the gut microbiota of *G. molesta* fed on an artificial diet (CK) and apples (A) was significantly different (PERMANOVA: R^2^ = 0.654, *p* = 0.001), but there was no significant difference between the artificial diet-feeding group (CK) and the plum-feeding group (PL) (PERMANOVA: R^2^ = 0.501, *p* = 0.101). Interestingly, there was no significant difference in the structure of the gut microbiota of *G. molesta* that fed on peaches (P) and nectarines (N) (PERMANOVA: R^2^ = 0.550, *p* = 0.101), but the difference between the peach-feeding group (P) and the peach shoot-feeding group (PS) was significant (PERMANOVA: R^2^ = 0.564, *p* = 0.001).

**Figure 6:**
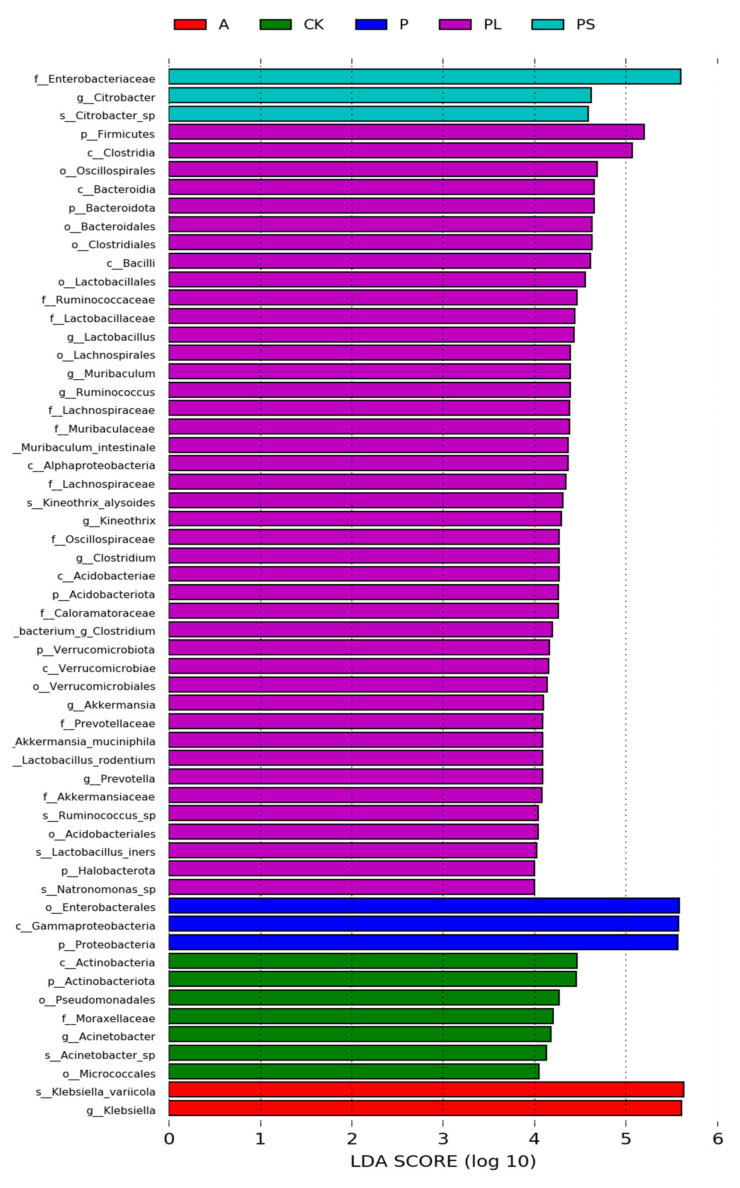
Bacterial taxa with linear discriminant analysis (LDA) score greater than four in the gut microbiota of *G. molesta* feeding on different host plants.

**Figure 7:**
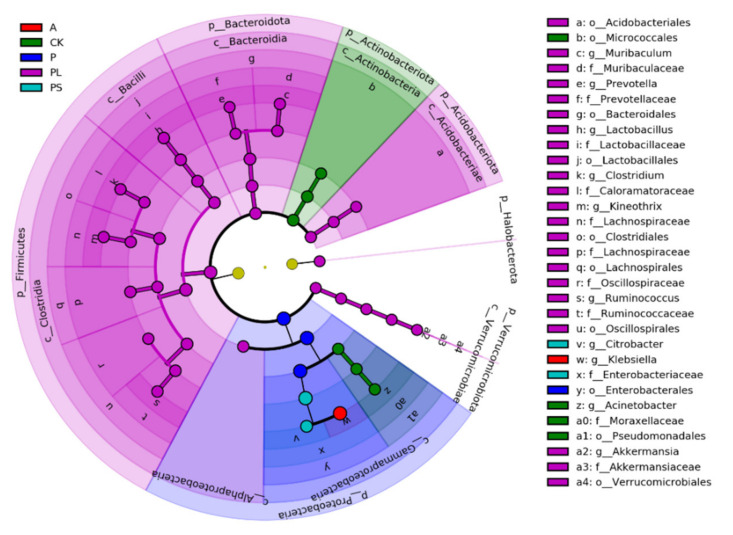
Cladogram of bacterial biomarkers, from the phylum (innermost ring) to genus (outermost ring) level, with an LDA score > 4. Differential bacterial taxa are marked by lowercase letters. Each small circle at different taxonomic levels represents a taxon at that level, and the diameter of the circle is proportional to the relative abundance. The coloring principle is to color the species with no significant difference as yellow, and the other different species as the group with the highest abundance of the species. Different colors represent different groups, and nodes with different colors represent the communities that play an important role in the group represented by the color.

In order to find biomarkers with statistical differences between different groups, we used linear discriminant analysis (LDA) effect size (LEfSe) to screen out different taxa at various levels (kingdom, phylum, class, order, family, genus, species) between different groups based on a standard LDA value greater than four (Figure 6). Meanwhile, we drew the cladogram from phylum to genus to fully understand the distribution of these different taxa at various taxonomic levels (Figure 7). In the plum-feeding group (PL), the gut microbiota of *G. molesta* had the most different taxa (LDA > 4). There were 34 taxa mainly concentrated in Firmicutes, Bacteroidota, Verrucomicrobiota, and Acidobacteriota. Six different taxa differed significantly in the gut microbiota of *G. molesta* feeding on an artificial diet (CK), three of them exclusive to Actinobacteriota and the other three belonging to Proteobacteria. Three and two taxa, mainly concentrated in the phylum Proteobacteria, were in the gut microbiota in the peach-feeding (P) and peach shoot-feeding (PS) groups, respectively. The dominant Enterobacteriaceae was also the most highly discriminating taxon in the peach shoot-feeding (PS) group. Finally, Klebsiella in the gut microbiota of the apple-feeding group (A) belonged to the phylum Proteobacteria. Overall, these analyses confirmed the effect of host diets on the gut community structure in *G. molesta*, similar to what was observed in other herbivores in Lepidoptera.

### 2.3. Functional Prediction of Gut Microbiota

**Figure 8:**
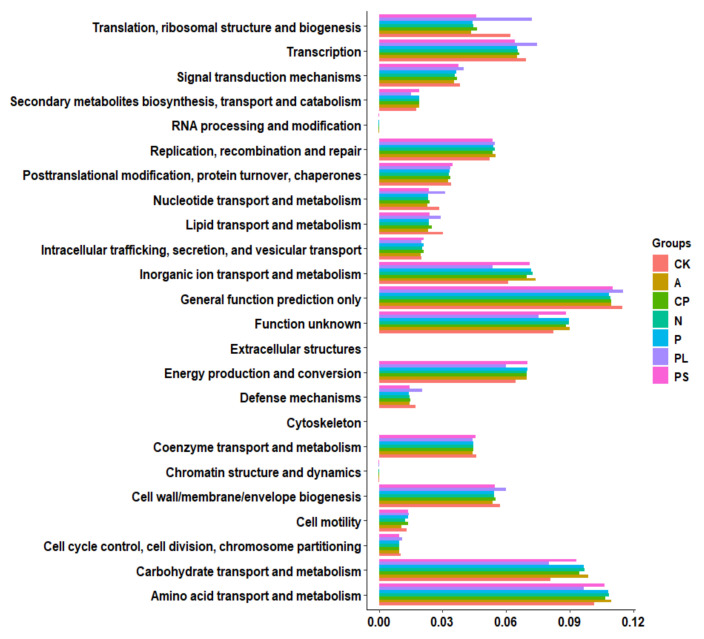
Comparison of predicted COG functions of gut bacteria of *G. molesta* feeding on different host plants.

In order to better understand the important role of the gut microbiota of *G. molesta*, we used PICRUSt2 software to predict the functional gene compositions of samples based on 16S rDNA sequencing data and compared them with the Cluster of Orthologous Groups (COG) database. The results showed that most functional prediction categories are related to metabolic and cellular processes. The main metabolic functions include amino acid transport and metabolism, carbohydrate transport and metabolism, inorganic ion transport and metabolism, energy production and conversion, transcription, replication, and recombination and repair and represent the most active functions inside the gut (Figure 8).

**Figure 9:**
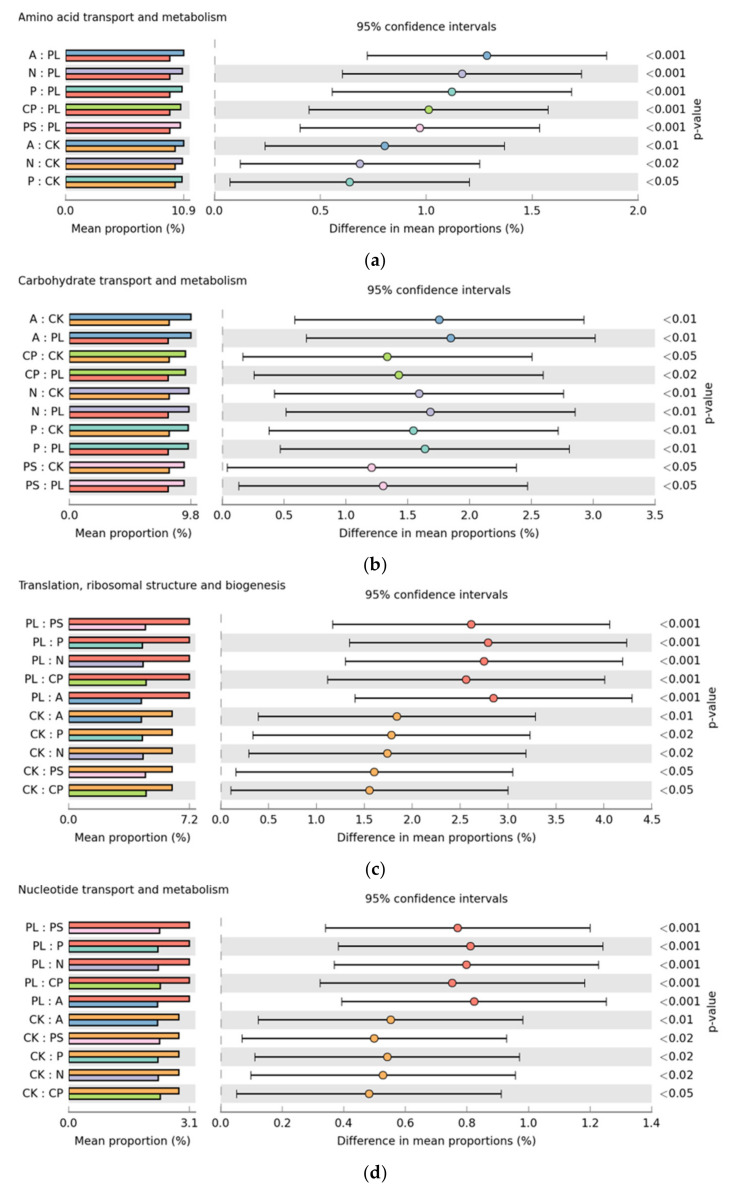
Significant differences (one-way ANOVA, Tukey–Kramer post-hoc test, *p* < 0.05.) in functions abundance of (**a**) Amino acid transport and metabolism, (**b**) Carbohydrate transport and metabolism, (**c**) Translation, ribosomal structure and biogenesis and (**d**) Nucleotide transport and metabolism.

As the largest functional prediction category, the plum-feeding group proved to have the lowest proportion of amino acid transport and metabolism (9.66 ± 0.35%) and differed from the apple-feeding (10.95 ± 0.02%), crisp pear-feeding (10.67 ± 0.05%), nectarine-feeding (10.83 ± 0.06), peach-feeding (10.78 ± 0.01%), and peach shoot-feeding (10.63 ± 0.15%) groups dramatically. The artificial diet-feeding group had the second lowest percentages (10.14 ± 0.20%), which were significantly less than the apple-feeding, nectarine-feeding, and peach-feeding groups (Figure 9a). Similarly, for carbohydrate transport and metabolism, the proportions in the apple-feeding (9.84 ± 0.01%), crisp pear-feeding (9.42 ± 0.12%), nectarine-feeding (9.68 ± 0.09), peach-feeding (9.63 ± 0.02%), and peach shoot-feeding (9.30 ± 0.36%) groups were significantly higher than in the artificial diet-feeding (8.09 ± 0.58%) and plum-feeding (7.99 ± 0.58%) groups (Figure 9b). Nevertheless, for translation, ribosomal structure, and biogenesis, the proportions in the plum-feeding (7.19 ± 0.89%) and artificial diet-feeding (6.18 ± 0.63%) groups were distinct from the apple-feeding (4.34 ± 0.01%), crisp pear-feeding (4.63 ± 0.15%), nectarine-feeding (4.44 ± 0.04), peach-feeding (4.40 ± 0.02%), and peach shoot-feeding (4.58 ± 0.20%) groups. The proportions were increased after *G. molesta* was transferred from the artificial diet to plums, but it was not significant (Figure 9c). For nucleotide transport and metabolism, the proportions were basically the same as for translation, ribosomal structure, and biogenesis (Figure 9d). These results indicate that the gut microbiota accelerated the transport and metabolism of nutriment after *G. molesta* was transferred from the artificial diet to natural plants, except plums, and enhanced the cellular processes and signaling and information processing after being transferred to plums.

## 3. Discussion

As a kind of fruit tree pest, which is seasonally transferred, *G. molesta* can damage different species of fruit trees of the Rosaceae family in the same or different generations in 1 year. We have studied the gut bacterial diversity and community composition of *G. molesta* larvae feeding on different host plants of the Rosaceae family and on artificial feed. The experimental results have given us a more comprehensive understanding of the relationship between *G. molesta* and its symbiotic microorganisms. According to our experimental data, we can preliminarily conclude that the diversity and abundance of the gut bacterial community of *G. molesta* are affected by the host diet. These results reveal a complex symbiotic community in the gut bacteria of *G. molesta* larvae and provide a theoretical basis for understanding the adaptation mechanism of *G. molesta* and its host plants. 

Previous studies have shown that the structure of insect gut bacterial communities can be affected by the host species and the host diet [7,31,34,35,36,37,38], and the influences of the host diet are much greater than those of the host species [39,40]. The diversity of gut bacteria has changed to varying degrees, with diversity increasing in plums and decreasing in the other four hosts after switching from the artificial feed to different host plants. Similar to the research that gut bacterial diversity in *P. xylostella* decreased after the host shift from radishes to peas, the structure of the gut bacteria varies with the diet [7].

At the phylum level, there were no significant differences in the bacterial composition of *G. molesta* across the five diets of apples, peaches, nectarines, crisp pears, and peach shoots, but the diversity of gut bacteria was significantly lower than that of the artificial feed and plums. In these five treatments, Proteobacteria was the absolute dominant phylum, and its relative abundances were more than 90%, similar to previous researches [20,31,33]. However, in the two treatment groups that fed on an artificial feed and plums, the following four phyla were dominant: Proteobacteria, Firmicutes, Bacteroidota, and Actinobacteriota. Many studies have reported that Proteobacteria and Firmicutes are the dominant bacterial phyla in insect gut bacterial communities, especially Lepidoptera [34,41,42,43,44]. They play a key role in carbohydrate metabolism, amino acid metabolism, and membrane transport pathways of the host [20,45,46]. Stably colonized gut bacteria, such as Proteobacteria, could be functionally crucial for insects to adapt to specific host plants [7]. Our research demonstrates this important role in host metabolic pathways and adaptation. Future research needs to combine metabolomics to explore how the gut bacteria affect host metabolic processes.

Enterobacteriaceae, which has been reported to appear in the gut bacteria of many insects [37,47,48], dominates almost every sample. Studies have shown that Enterobacteriaceae not only plays an important role in the host’s sugar metabolism and protects against parasites and pathogens in the insect gut [49], but also contributes to courtship, reproduction, and even degradation of polyethylene [13,31,50]. It has been reported that the high abundance of Enterobacteriaceae may be related to host adaptability, suggesting the importance of Enterobacteriaceae in the fitness of *G. molesta* [4,31]. The most abundant genus in Enterobacteriaceae is *Klebsiella*, which occurs widely in the guts of Lepidoptera and other herbivores and is a potentially beneficial, nonpathogenic microbe [51,52,53]. *Klebsiella* can degrade cellulose and sugarcane residues in *Diatraea saccharalis* [54,55], provide usable nitrogen for apple maggot flies, increase immunity in *Ceratitis capitata*, and promote the development of larvae [8,14,56,57].

*Lactobacillus* and *Clostridium* were also detected in our study with high relative abundance in the artificial feed (CK) and plum (PL) diets, similar to *Enterococcus* in the crisp pear-feeding (CP) group and *Acinetobacter*, *Bacillus*, and *Corynebacterium* in the artificial diet (CK) group. These results suggest that the content of the gut microbiota varies by host plants, even when they are in the same family Rosaceae. Gut bacterial composition shifts qualitatively and quantitatively according to the host’s functional needs [7]. *Enterococcus* has been found to be the most common gut bacteria in Lepidoptera, such as *Spodoptera littoralis*, *Busseola fusca*, *Helicoverpa armigera*, and *Brithys crini* [51,58,59,60]. As a kind of insect symbiotic bacteria that can be stably maintained during metamorphosis [61], *Enterococcus* was reported to confer a protection against pathogens [10], fixing toxic molecules of the plants [62], increasing host fitness [9], and increasing the tolerance of a toxic diet [60].

PCoA analysis revealed a distinct difference in the compositions of gut bacterial communities in the larvae of *G. molesta* (PERMANOVA: R^2^ = 0.787, *p* = 0.001). We found that the structure of the gut microbiota of *G. molesta* fed on the artificial diet (CK) and apples (A) was significantly different (PERMANOVA: R^2^ = 0.654, *p* = 0.001), but there was no significant difference between the artificial diet-feeding group (CK) and the plum-feeding group (PL) (PERMANOVA: R^2^ = 0.501, *p* = 0.101). One possible conjecture to explain this is that plum and artificial feeds have similar nutritional components and contain more amino acids and nutrients required for the growth of *G. molesta*, which can thus better meet the growth needs of *G. molesta*. The secondary metabolites in plants and the nutrient requirements of the host affect the composition of the gut microbiota [7]. Furthermore, previous studies have shown that *G. molesta* feeding on an artificial diet produces more eggs when they feed on host plums than when they feed on apples and peaches [17]. The previous research in our laboratory also found that, among the host species of apples (Gala, Qinguan), begonias, peaches, nectarines, and plums, the finite rate of increase (λ) of *G. molesta* on plum was the largest and the population doubling time (t_d_) was the shortest (unpublished). Therefore, we hypothesized that plums might be the most suitable host for *G. molesta*. However, the host selection of insects is affected by many factors, such as environment, maturity, and volatiles of host plants, so this hypothesis needs to be further confirmed. Interestingly, there was no significant difference in the structure of the gut microbiota of *G. molesta* fed on peaches (P) and nectarines (N) (PERMANOVA: R^2^ = 0.550, *p* = 0.101), but that of the peach-feeding group (P) and the peach shoot-feeding group (PS) was significantly different (PERMANOVA: R^2^ = 0.564, *p* = 0.001), indicating that the gut microbiota of *G. molesta* that eats fruits of different species are more similar, while there are significant distinctions between those of *G. molesta* eating fruits and shoots from the same species. An alternative explanation may be that the gut microbiota are influenced by the nature of the host plant. There was also evidence that the change in gut microbiota is a gradual process [7]. In our study, only one generation was observed after host transference, which is a limitation. Observing the composition of *G. molesta* gut microbiota in successive generations will help us understand the changes in *G. molesta* gut microbiota in host metastasis better in the future. At the same time, the structure and important functions of gut microbiota in the adaptability of different Rosaceae hosts should be explored by combining metabonomics and metagenomics.

In conclusion, our results confirmed that the gut bacterial structure of *G. molesta* can be influenced by the host plant. After *G. molesta* feeding on an artificial diet was transferred to plums, the diversity increased and the relative abundances of Firmicutes and Bacteroidota increased significantly. After being transferred to apples, crisp pears, peaches, nectarines, and peach shoots, the diversity decreased and the relative abundance of Proteobacteria increased prominently. Gut bacterial communities were influenced by host diets and may play an important role in host adaptation. However, there were still many limitations in our study. Future experiments should be designed to observe the changes in gut bacterial communities in *G. molesta* adaptation to different hosts for successive generations, and the important functions of gut bacterial communities in host adaptation should be elucidated with the methods of multi-omics, so as to find new targets for controlling fruit tree pests. Our study provides a theoretical basis for the study of gut symbiotic bacteria in *G. molesta*.

## 4. Materials and Methods

### 4.1. Insect Rearing and Sample Processing 

Eggs used in this experiment were taken from the *G. molesta* colony, which was maintained on an artificial diet for more than 6 years at 26 ± 0.5 °C 70 ± 10% relative humidity (RH) with a photoperiod (L:D) of 15 h:9 h in the Integrated Pest Management Laboratory of Northwest A&F University in Yangling, Shaanxi, China. Newly hatched larvae were reared by an artificial diet (CK), fresh Gala apples (A), crisp pears (CP), nectarines (N), peaches (P), peach shoots (PS), and plums (PL) until the fourth instar. All host plants were collected during the swelling period from the Economic Arboretum, Northwest A&F University, in July 2020. All samples were collected for this experiment from the fourth-instar larvae.

### 4.2. DNA Extraction 

The larvae were selected and surface-sterilized in 75% ethanol for 60 s, followed by 3 washes in sterile water for 30 s. Midgut tissues were dissected with sterile forceps in a sterile petri dish with a diameter of 90 mm and rinsed again in sterile phosphate buffered saline (PBS) [7,20,31,33]. Fifty midguts were collected per sample using a sterile centrifuge tube. Each treatment had 3 replicates. The total DNA of all samples was extracted by using the MN NucleoSpin 96 Soil DNA kit (MACHEREY-NAGEL, Düren, Germany). DNA quantity and quality were measured on a NanoDrop 2000 spectrophotometer (Thermo Fisher Technology (China) Co., Ltd., Shanghai, China). The total DNA was preserved at −80 °C.

### 4.3. PCR Amplification and High-Throughput Sequencing 

The universal primer pair, 338F (5′-ACTCCTACGGGAGGCAGCA-3′) and 806R (5′-GGACTACHVGGGTWTCTAAT-3′), with a sequencing adapter at the end, were used to amplify the V3-V4 hypervariable region of the bacterial 16s rDNA gene. First-round tailed polymerase chain reaction (PCR) amplification was performed in a volume of 10 µL with the following reaction components: 50 ng of genome DNA, 1 μL of 10 μM primer F, 0.3 μL of the 10 μM primer R, 5 μL of KOD FX Neo Buffer, 2 μL of dNTP, and 0.2 μL of KOD FX Neo. PCR cycling parameters were 95 °C for 5 min, followed by 25 amplification cycles of 95 °C for 30 s, 50 °C for 30 s, and 72 °C for 40 s, with a 7 min final extension at 72 °C. In order to add indices and adapter sequences, the second-round tailed PCR amplification was performed under the following conditions: 98 °C for 30 s, followed by 10 cycles of 98 °C for 10 s, 65 °C for 30 s, 72 °C for 30 s, and a 5 min final extension at 72 °C. The reaction system was a volume of 20 μL comprised of 5 μL for the products of first-round PCR, 2.5 μL of MPPI-a, 2.5 μL of MPPI-b, and 10μL of 2 × Q5 HF MM. The PCR products were detected on 1.8% agarose gels and purified, quantified, and homogenized to form a sequencing library. Qualified libraries were sequenced using Illumina HiSeq 2500 by Biomarker Co., Ltd. (Beijing, China).

### 4.4. Statistical and Bioinformatics Analysis

A total of 1,531,209 paired-end reads were generated to survey the bacterial communities. The raw data were first analyzed using Trimmomatic (version 0.33, Golm, Germany). We then used cutadapt (version 1.9.1, TU Dortmund, Germany) software to identify and remove primer sequences for high-quality reads that did not contain primer sequences. The high-quality reads of each sample were spliced through overlap by FLASH (version 1.2.7, Baltimore, MD, USA) software, and the resulting splicing sequence was clean reads. We used UCHIME (version 4.2, http://drive5.com/usearch/manual/uchime_algo.html (accessed on 24 June 2021)) for identifying and removing the chimera sequence and obtained the final valid data as effective reads. According to the similarity of the sequence, the effective sequence was classified into multiple operational taxonomic units (OTUs) at the similarity level of 97% through the software USEARCH (version 10.0, http://drive5.com/usearch/ (accessed on 24 June 2021)), and the OTUs were filtered by 0.005% of the sequence numbers of all sequences. All representative sequences were annotated and blasted against Silva database version 123 (http://www.arb-silva.de (accessed on 24 June 2021)) using RDP Classifier (https://sourceforge.net/projects/rdpclassifier/ (accessed on 24 June 2021)) with a confidence threshold at 80%. Principal coordinate analysis (PCoA) based on the Bray−Curtis distance was applied to reveal the differences in bacterial communities between groups. Permutational multivariate analysis of variance (PERMANOVA) was performed for pairwise comparison. Here, the Bray−Curtis distance was used as a metric of similarity between the bacterial communities based on the abundance of OTUs between samples. Linear discriminant analysis (LDA) was used to screen the biomarkers for statistical differences between different groups with LDA scores greater than 4. A cladogram was drawn to show the distribution of these biomarkers at different taxonomic levels by Galaxy (http://huttenhower.org/galaxy/ (accessed on 24 June 2021)). Microbiota functions were predicted by annotating pathways of OTUs against the COG database using PICRUSt2. We used extended error bars in STAMP software (https://beikolab.cs.dal.ca/software/STAMP (accessed on 24 June 2021)) to show the significant differences in several functions at the second level of Cluster of Orthologous Groups (COG) between different groups.

Differences were considered significant when *p* < 0.05 and extremely significant when *p* < 0.01. SPSS 26.0 (https://www.ibm.com/products/spss-statistics (accessed on 24 June 2021)) was used for statistical analysis.

## Figures and Tables

**Table 1:** Summary of sequence statistics for the Illumina HiSeq runs of all samples.

Sample ID	Raw Reads	Clean Reads	Effective Reads	AvgLen (bp)	GC (%)	Effective (%)
A1	79884	67772	67538	429	56.69	84.55
A2	79835	67914	67618	429	56.71	84.70
A3	73610	62130	61705	429	56.69	83.83
CK1	79980	70117	64592	418	54.89	80.76
CK2	80062	70018	67177	423	55.41	83.91
CK3	80384	69886	67041	423	55.73	83.40
CP1	80015	67814	67101	429	56.59	83.86
CP2	79585	68569	66419	426	56.53	83.46
CP3	79957	68144	66329	426	56.58	82.96
N1	44803	36668	36231	429	56.5	80.87
N2	56666	36618	35694	429	56.49	62.99
N3	69729	56067	55246	429	56.67	79.23
P1	79923	67884	67687	429	56.60	84.69
P2	63619	53983	53828	429	56.62	84.61
P3	77451	65509	65201	429	56.59	84.18
PL1	80101	66639	62056	413	55.01	77.47
PL2	79776	66332	61239	413	55.22	76.76
PL3	80136	68242	64313	421	55.69	80.25
PS1	62578	52190	51917	429	56.58	82.96
PS2	64642	54192	53968	429	56.59	83.49
PS3	58473	49052	48669	427	56.47	83.23

Raw reads represent the number of original reads sequenced by Illumina HiSeq. Clean reads are the number of high-quality reads obtained after quality control and splicing. Effective reads indicate the number of effective sequences with non-chimeras. AvgLen (bp) is the average sequence length of all samples. GC (%) is the percentage of G and C type bases in the total base. Effective (%) is the percentage of effective reads in raw reads.

**Table 2:** The relative abundance of the top four phyla.

Sample ID	Proteobacteria	Firmicutes	Bacteroidota	Actinobacteriota
CK	59.18 ± 13.26 b	17.74 ± 4.85 b	8.32 ± 2.56 b	7.85 ± 3.79 a
A	99.43 ± 0.07 a	0.27 ± 0.05 c	0.08 ± 0.01 c	0.11 ± 0.05 b
CP	94.34 ± 2.32 a	3.02 ± 1.28 c	0.20 ± 0.10 c	0.67 ± 0.23 b
N	98.13 ± 0.60 a	0.96 ± 0.24 c	0.57 ± 0.34 c	0.07 ± 0.01 b
P	99.52 ± 0.12 a	0.28 ± 0.16 c	0.07 ± 0.01 c	0.09 ± 0.04 b
PL	32.75 ± 18.59 b	32.92 ± 8.90 a	15.06 ± 4.06 a	2.80 ± 0.86 b
PS	95.63 ± 2.93 a	0.30 ± 0.07 c	0.12 ± 0.02 c	0.03 ± 0.00 b

Data are the mean ± SE. The a, b and c indicate the significant differences in relative abundance in the same column in the mean values. The same lowercase letter indicates that these is no significant difference between the groups, and different letters indicate that the differences between the groups is significant (one-way ANOVA, Tukey post-hoc test, *p* < 0.05).

**Table 3:** The relative abundance of the top 10 families.

Sample ID	*Enterobacteriaceae*	*Lachnospiraceae*	*Ruminococcaceae*	*Lactobacillaceae*	*Muribaculaceae*	*Oscillospiraceae*	*Caloramatoraceae*	*Moraxellaceae*	*Prevotellaceae*	*Akkermansiaceae*
CK	47.43 ± 16.05 b	2.79 ± 0.46b	2.03 ± 0.37 b	2.14 ± 0.49 b	2.60 ± 0.72 b	1.33 ± 0.37 b	1.24 ± 0.56 b	3.84 ± 0.76 a	1.10 ± 0.38 b	0.61 ± 0.08 b
A	99.15 ± 0.12 a	0.06 ± 0.01b	0.04 ± 0.01 b	0.03 ± 0.01 c	0.03 ± 0.01 c	0.03 ± 0.01 c	0.02 ± 0.01 b	0.01 ± 0.00 b	0.04 ± 0.00 c	0.02 ± 0.00 b
CP	92.00 ± 2.71 a	0.42 ± 0.20b	0.21 ± 0.11 b	0.29 ± 0.06 c	0.07 ± 0.04 c	0.18 ± 0.09 c	0.19 ± 0.09 b	0.05 ± 0.02 b	0.02 ± 0.01 c	0.03 ± 0.01 b
N	96.43 ± 1.04 a	0.19 ± 0.02b	0.11 ± 0.02 b	0.10 ± 0.01 c	0.06 ± 0.02 c	0.07 ± 0.01 c	0.05 ± 0.01 b	0.00 ± 0.00 b	0.04 ± 0.00 c	0.04 ± 0.00 b
P	98.72 ± 0.40 a	0.03 ± 0.01b	0.02 ± 0.00 b	0.02 ± 0.00 c	0.02 ± 0.00 c	0.02 ± 0.00 c	0.01 ± 0.00 b	0.01 ± 0.00 b	0.01 ± 0.00 c	0.01 ± 0.00 b
PL	23.54 ± 21.21 b	8.97 ± 2.53a	5.86 ± 1.71 a	5.21 ± 1.21 a	4.59 ± 1.30 a	3.53 ± 0.87 a	3.50 ± 0.94 a	0.41 ± 0.15 b	2.39 ± 0.62 a	2.41 ± 0.69 a
PS	95.35 ± 3.07 a	0.08 ± 0.02b	0.06 ± 0.01 b	0.04 ± 0.01 c	0.03 ± 0.01 c	0.03 ± 0.01 c	0.03 ± 0.01 b	0.00 ± 0.00 b	0.03 ± 0.01 c	0.01 ± 0.00 b

Data are the mean ± SE. The a, b and c indicate the significant differences in relative abundance in the same column in the mean values. The same lowercase letter indicates that these is no significant difference between the groups, and different letters indicate that the differences between the groups is significant (one-way ANOVA, Tukey post-hoc test, *p* < 0.05).

## Data Availability

The data presented in this study are available in supplementary material here.

## Supplementary Materials

The following are available online at https://www.mdpi.com/article/10.3390/ijms22136843/s1, Tables S1–S5: Supporting data sets.
